# Exploring potential drug−drug interactions between masculinizing hormone therapy and oral pre‐exposure prophylaxis (F/TDF and F/TAF) among transgender men (iMACT study): a randomized, open‐label pharmacokinetic study in Thailand

**DOI:** 10.1002/jia2.26445

**Published:** 2025-04-07

**Authors:** Akarin Hiransuthikul, Narukjaporn Thammajaruk, Stephen Kerr, Rena Janamnuaysook, Siriporn Nonenoy, Piranun Hongchookiat, Rapee Trichavaroj, Yardpiroon Tawon, Jakkrapatara Boonruang, Nipat Teeratakulpisarn, Tim R. Cressey, Peter L. Anderson, Nittaya Phanuphak, the iMACT study team

**Affiliations:** ^1^ Institute of HIV Research and Innovation (IHRI) Bangkok Thailand; ^2^ Department of Preventive and Social Medicine Faculty of Medicine Chulalongkorn University Bangkok Thailand; ^3^ Biostatistics Excellence Centre Faculty of Medicine Chulalongkorn University Bangkok Thailand; ^4^ HIV‐NAT, Thai Red Cross AIDS Research Centre Bangkok Thailand; ^5^ The Kirby Institute University of New South Wales Sydney New South Wales Australia; ^6^ Center of Excellence in Transgender Health (CETH) Faculty of Medicine Chulalongkorn University Bangkok Thailand; ^7^ AMS/PHPT Research Collaboration Faculty of Associated Medical Sciences Chiang Mai University Chiang Mai Thailand; ^8^ Department of Pharmaceutical Sciences University of Colorado, Anschutz Medical Campus Aurora Colorado USA

**Keywords:** drug−drug interactions, masculinizing hormone therapy, pre‐exposure prophylaxis, Thailand, HIV prevention, transgender men

## Abstract

**Introduction:**

Concerns regarding potential drug−drug interactions (DDIs) between hormone therapy and pre‐exposure prophylaxis (PrEP) may hinder PrEP use among transgender persons. Transgender men have often been overlooked in biomedical HIV research, and potential DDIs between masculinizing hormone therapy (MHT) and PrEP have not been addressed. We aimed to assess the potential DDIs between MHT and daily oral PrEP among transgender men.

**Methods:**

Transgender men without HIV who never underwent oophorectomy were enrolled between May and October 2022. Participants were randomly assigned to receive emtricitabine‐tenofovir disoproxil fumarate (F/TDF) or emtricitabine‐tenofovir alafenamide (F/TAF) for daily oral PrEP. Intramuscular testosterone enanthate 200 mg was administered every 2 weeks from baseline to week 12, while oral PrEP was initiated at week 6 and continued until week 16. Pharmacokinetic (PK) sampling was conducted at weeks 4 and 12 to assess the impact of PrEP on MHT and at weeks 12 and 16 to evaluate the impact of MHT on PrEP. Plasma total testosterone, TAF, tenofovir (TFV) and FTC; tenofovir‐diphosphate (TFV‐DP) and emtricitabine‐triphosphate (FTC‐TP) concentrations in peripheral blood mononuclear cells (PBMCs) were measured in all participants. Cervical and rectal tissues were obtained in a subset of 20 participants (10 per group) to measure TDF‐DP and FTC‐TP.

**Results:**

Thirty‐nine participants (19 F/TDF and 20 F/TAF) completed the PK visits. No significant changes in the PK parameters of plasma total testosterone, TFV, FTC and TAF (for F/TAF group); urine TFV and FTC; and PBMC and rectal tissue TFV‐DP and FTC‐TP were observed when MHT and PrEP were administered together. Both TFV‐DP and FTC‐TP concentrations in cervical tissue were significantly lower when MHT was co‐administered with F/TAF (TFV‐DP: median [IQR] of 12.9 [6.78–14.56] fmol/mg at weeks 12 vs. 20.63 [7.47–53.43] fmol/mg at week 16, *p* = 0.04; and FTC‐TP: 67.05 [27.24–77.24] fmol/mg vs. 120.43 [65.98–245.76] fmol/mg, *p* = 0.02).

**Conclusions:**

Our findings across multiple anatomical compartments suggest that oral PrEP should not affect the effectiveness of MHT and that F/TDF‐based PrEP should be effective when taken with MHT. However, further research is needed to assess the effectiveness of TAF‐based PrEP in transgender men.

**Clinical Trial Number:**

NCT04593680.

## 1 INTRODUCTION

Transgender persons face a heightened risk of HIV acquisition compared to the cisgender population. In the United States, the HIV prevalence among transgender men and transgender women were approximately 3.2% and 14.1%, respectively, compared to 0.4% among the general population [1]. Despite these data, transgender persons, particularly transgender men, have been largely overlooked in biomedical HIV research. Among transgender men at risk of sexually transmitted infections (STIs), only about 48.3% undergo HIV testing, and 10.2% of those tested who are at risk for STIs actually received pre‐exposure prophylaxis (PrEP) [1]. Numerous factors contribute to the low uptake of PrEP among transgender persons, with one of the major concerns being the potential drug−drug interactions (DDIs) between gender‐affirming hormone therapy (GAHT) and PrEP [2, 3]. Unfortunately, information on this important issue remains extremely limited among transgender men.

Masculinizing hormone therapy (MHT) is widely used among transgender men to develop secondary sex characteristics aligned with their affirmed gender and to diminish the sex characteristics of sex assigned at birth [4, 5]. This usually involves the use of testosterone, available in various forms such as injections, gels, patches, pellets implantation and oral formulations.

To address concern regarding potential DDIs between GAHT and PrEP, several studies have been conducted and published in recent years [6, 7, 8, 9, 10, 11, 12, 13, 14]. Unfortunately, only three small studies have included transgender men [10, 12, 13]. Using emtricitabine‐tenofovir disoproxil fumarate (F/TDF)‐based PrEP in dried blood spots (DBS) as a measure of PrEP drug adherence, findings suggest that clinically significant DDIs between MHT and F/TDF are not anticipated.

Tenofovir alafenamide (TAF), a novel prodrug formulation of tenofovir (TFV), provides higher intracellular concentrations of tenofovir‐diphosphate (TFV‐DP), the active intracellular metabolite of TFV in lymphatic tissue, while maintaining lower plasma TFV concentrations compared to TDF, mitigating the risk of bone and kidney adverse events [15]. Daily oral F/TAF has proven effective for HIV prevention in cisgender men who have sex with men and transgender women [16], but data on persons assigned female at birth, including transgender men, remain limited. Recent studies suggest the potential of F/TAF for HIV prevention among cisgender women, based on TFV‐DP concentrations in peripheral blood mononuclear cells (PBMCs) and vaginal tissue [17]. However, it is important to consider that MHT can affect the female reproductive organs, potentially increasing susceptibility to HIV acquisition.

This study aimed to assess potential DDIs between MHT and daily oral PrEP (F/TDF and F/TAF) in transgender men, utilizing pharmacokinetic (PK) measurements from various anatomical compartments. Our findings are important to provide evidence‐based guidance for PrEP utilization among transgender men.

## 2 METHODS

### 2.1 Enrolment and study population

The iMACT study prospectively enrolled 40 transgender men at the Tangerine Community Health Center, Institute of HIV Research and Innovation, in Bangkok, Thailand between May and October 2022. Eligible participants were Thai transgender men without HIV, aged 18–60 years, with body mass index (BMI) of 18.5−29.9 kg/m^2^, calculated creatinine clearance (CrCl) ≥60 ml/minute using Cockcroft‐Gault equation and alanine aminotransferase (ALT) ≤2.5 x upper limit of normal. Exclusion criteria included a history of allergy to the study hormonal compounds, who had undergone oophorectomy, had positive urine pregnancy test, had received injectable MHT in the previous 3 months, currently had hepatitis B or C, were currently using a medication with possible DDIs with MHT and/or PrEP, had a history of myocardial infarction or coronary artery disease or had history of gastrointestinal tract surgery that alter gastrointestinal tract were excluded. The study (clincaltrials.gov identifier: NCT04593680) received approval from the institutional review board of the Faculty of Medicine, Chulalongkorn University, Bangkok, Thailand (IRB No. 727/63), and all participants provided informed consent before undergoing any study procedures.

### 2.2 Study drugs

All participants received intramuscular testosterone enanthate 200 mg (Testoviron Depot®, Bayer, Leverkusen, Germany) every 2 weeks for MHT and either daily oral F/TDF (Truvada®, Gilead Sciences, Foster City, CA, USA) or F/TAF (Descovy®, Gilead Sciences) for PrEP.

### 2.3 Study procedures

**Figure 1:**
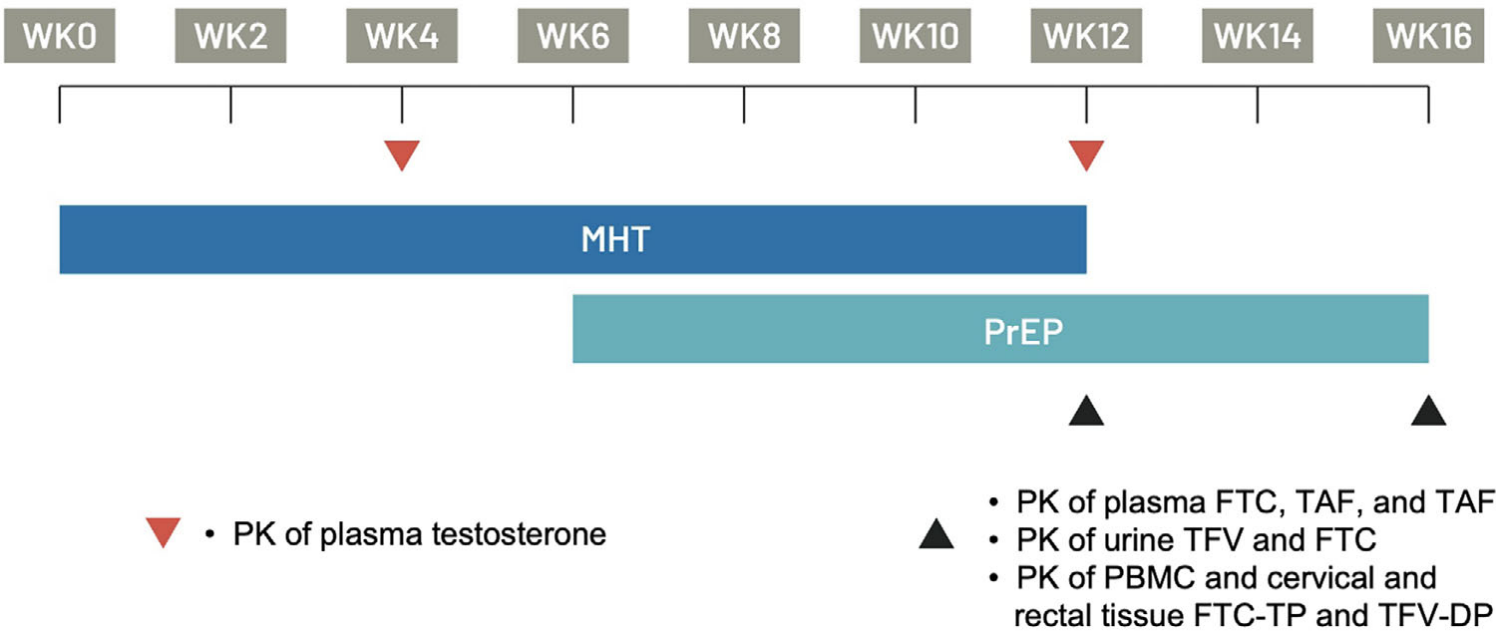
iMACT study scheme. MHT was initiated at week 0 until week 12. Daily PrEP was initiated at week 6 and continued without interruption until the completion of study period at week 16. Intensive PK parameters of total testosterone were assessed at week 4 (MHT only) and week 12 (MHT + PrEP). Intensive PK parameters of ARVs were assessed at week 12 (MHT + PrEP) and week 16 (PrEP only). Abbreviations: ARVs, antiretrovirals; MHT, masculinizing hormone therapy; PBMC, peripheral blood mononuclear cell; PK, pharmacokinetic; PrEP, pre‐exposure prophylaxis.

Participants were randomly assigned (1:1) to receive either F/TDF or F/TAF using block randomization. Both participants and the study team were aware of the participants’ assigned randomization. To assess the impact of PrEP on MHT and vice versa, PK assessments were performed at specific study periods when participants received (1) MHT alone, (2) MHT and PrEP, and (3) PrEP alone, in chronological order, with each participant serving as their own control. The intervals between study drug administrations were based on each drug's half‐life to ensure steady‐state conditions were achieved and the prior drug was fully eliminated. MHT was administered at study entry and every 2 weeks until week 12, while PrEP was initiated at week 6 and prescribed until the end of study at week 16 (Figure 1). The sequence used in this study was selected to prioritize consistent PrEP use and avoiding any temporary discontinuation. All participants understood the study process, including the planned interruption of MHT and the initial PrEP‐free period. During study visits, staff informed participants about the potential effects of MHT interruption, particularly the possibility of recurring menstruation. While other major reversals of MHT effects were considered less likely due to the short duration of both MHT use and the interruption [18], participants were made aware of this possibility. Throughout the study, participants received counselling on the consistent use of condoms to prevent pregnancy and reduce the risk of HIV acquisition, with particular emphasis during the PrEP‐free period.

The first intensive PK assessment for total testosterone was performed at week 4 to assess total testosterone without PrEP. At week 12, the second intensive assessment assessed total testosterone and PrEP PK parameters in the presence of MHT and PrEP. MHT was then discontinued. The third intensive PK assessment was conducted at week 16 to assess PrEP PK parameters without MHT. The comparison between week 4 and week 12 provided insights into the potential impact of PrEP on MHT, while the comparison between week 12 and week 16 provided information on the potential impact of MHT on PrEP.

To ensure adherence to daily oral PrEP, electronic directly observed therapy was performed in addition to pill counts and self‐reported use at each visit. The study staff contacted participants daily, either through video calls or messaging applications, to confirm their adherence to the study drug.

### 2.4 PK methods

A 14‐day PK study was performed for plasma total testosterone, and a 24‐hour PK study was performed for the components of PrEP. During the PK visits for plasma testosterone at week 4 and week 12, plasma samples were collected at t = 0 (pre‐dose), 0.5, 1, 1.5, 2, 2.5, 3, 4, 7 and 14 days. For the PK visits of PrEP drug components at week 12 and week 16, plasma samples were collected at t = 0 (pre‐dose), and then at 0.5, 1, 2, 4, 6, 8, 10, 12 and 24 hours after directly observed ingestion of study drugs with a standardized meal. The focus was on time points close to the initial dosing to accurately capture the peak concentrations of each study drug. PBMC samples were collected at t = 2 (C_2_) and 24 (C_24_) hours and all urine passed during the entire 24‐hour period was collected. Cervical and rectal tissues were obtained at the PK visit 16–24 hours post‐dose in a subset of 20 participants (10 per group). Tissue pinch biopsies were weighed, flash frozen at −70°C and stored for analysis using a validated assay. The lower limit of quantification was 0.025 ng/ml for plasma total testosterone, 2 ng/ml for plasma TAF, 5 ng/ml for plasma TFV and FTC, 100 ng/ml for urine TFV, 1000 ng/ml for urine FTC, 200 fmol/sample for TFV‐DP in PBMCs, 500 fmol/sample for FTC‐TP in PBMCs, 25 fmol/sample for TFV‐DP in mucosal tissues and 0.1 pmol for FTC‐TP in mucosal tissues. Detailed specimen collection and processing procedures are available in the Supplementary File.

### 2.5 Statistical analysis

Statistical and PK analyses were performed using Stata/SE 17.0 (StataCorp, College Station, TX, USA). The area under plasma concentration time curve from time zero to 14 days for total testosterone (AUC_last_), the area under plasma concentration curve from time zero to 24 hours (AUC_0−24_) for PrEP drug components and the maximum concentration (C_max_) were derived using a non‐compartmental analysis. The geometric mean (GM) and % coefficient of variation (%CV) were summarized at each study week. Generalized estimating equations were used to assess changes in the geometric mean of AUC_last_, AUC_0−24_ and C_max_ to calculate the geometric mean ratio (GMR) for plasma total testosterone, TAF, TFV and FTC; urine TFV and FTC; and PBMC TFV‐DP and FTC‐TP. The reference week was the point at which either MHT or PrEP was administered alone. Cervical and rectal tissues TFV‐DP and FTC‐TP were presented as median (IQR) at each study week and compared with a Wilcoxon sign‐rank test. In accordance with the US FDA Guidelines for Industry on Drug Interaction Studies, the 90% confidence intervals around the GMR were calculated.

## 3 RESULTS

A total of 39 transgender men were included in this analysis: 19 in the F/TDF group and 20 in the F/TAF group. One participant withdrew from the study due to personal reasons, and was excluded from the analysis. The demographic characteristics of the 39 participants are summarized in Table 1. None of the participants had a hysterectomy. Based on all measurements, all participants had 100% adherence to MHT and PrEP per the study protocol.

**Table 1:** Participants’ baseline characteristics

Characteristics	F/TDF group (*N* = 19)	F/TAF group (*N* = 20)
Age, years	34 (28−39)	28 (23−33)
Weight, kg	61.2 (55.7−68.0)	57.7 (51.2−63.5)
Height, cm	163 (160−169)	158 (153−161)
Body mass index, kg/m^2^	22.1 (21.3−24.7)	23.8 (20.5−25.2)
CrCl, ml/minute	124.9 (105.1−138.5)	123.7 (102.0−145.8)
ALT, IU/l	12 (11−17)	15 (10−19)

*Note*: Data are presented in median (IQR).

Abbreviations: ALT, alanine aminotransferase; CrCl, creatinine clearance.

### 3.1 Plasma total testosterone

**Figure 2:**
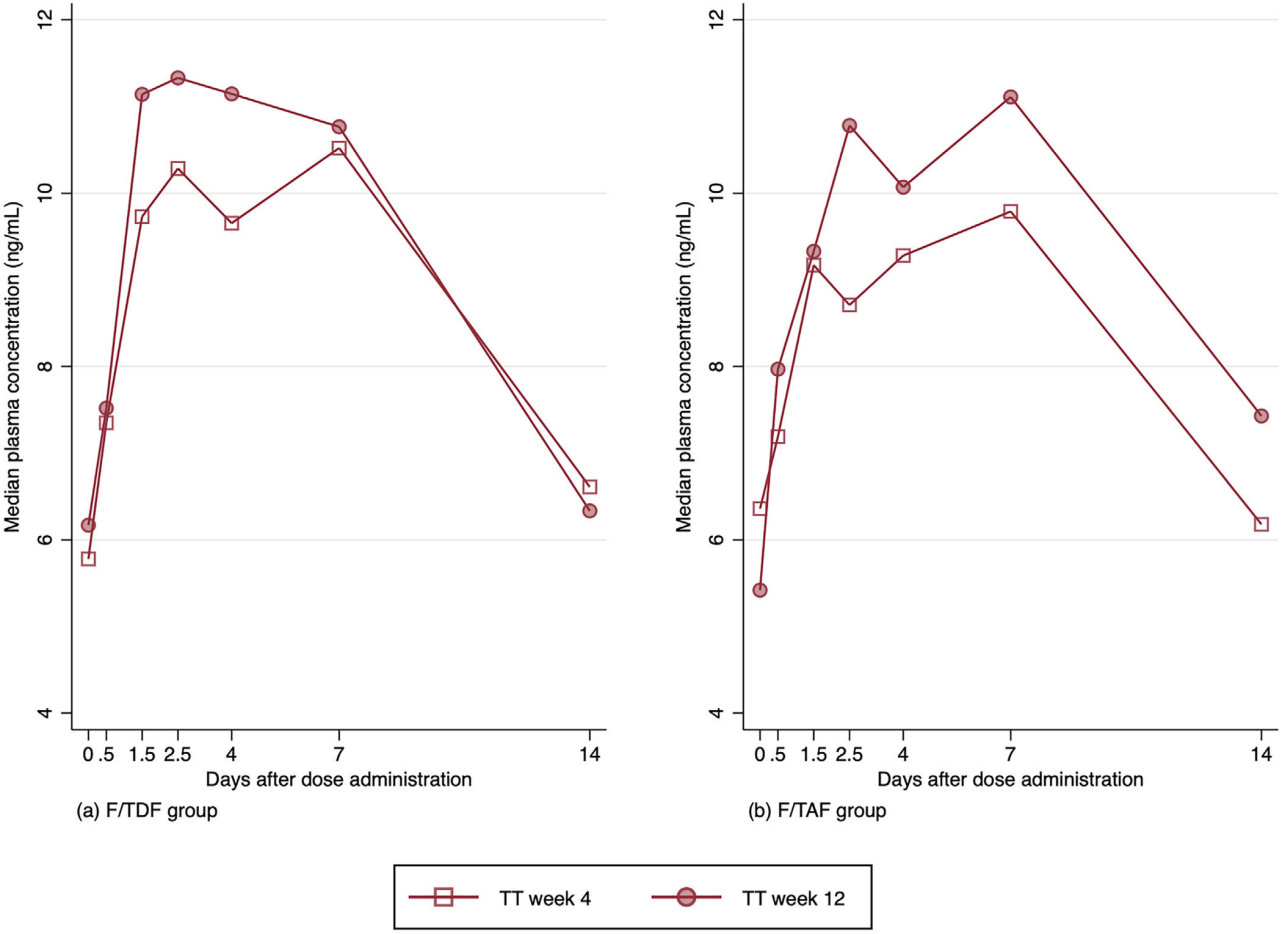
Median concentration times curves of plasma testosterone at week 4 (without PrEP) and week 12 (with PrEP): (a) F/TDF group and (b) F/TAF group. Abbreviation: TT, total testosterone.

The GMRs for AUC_last_ and C_max_ at weeks 4 (reference) and weeks 12 for plasma total testosterone were as follows: F/TDF group, 1.09 (0.97−1.23, *p* = 0.29) and 1.09 (0.99−1.20, *p* = 0.24); and F/TAF group, 1.10 (1.02−1.17, *p* = 0.06) and 1.10 (0.99−1.21, *p* = 0.19), respectively. The median plasma concentration‐time profiles of total testosterone are shown in Figure 2.

### 3.2 Plasma TAF, TFV and FTC

**Figure 3:**
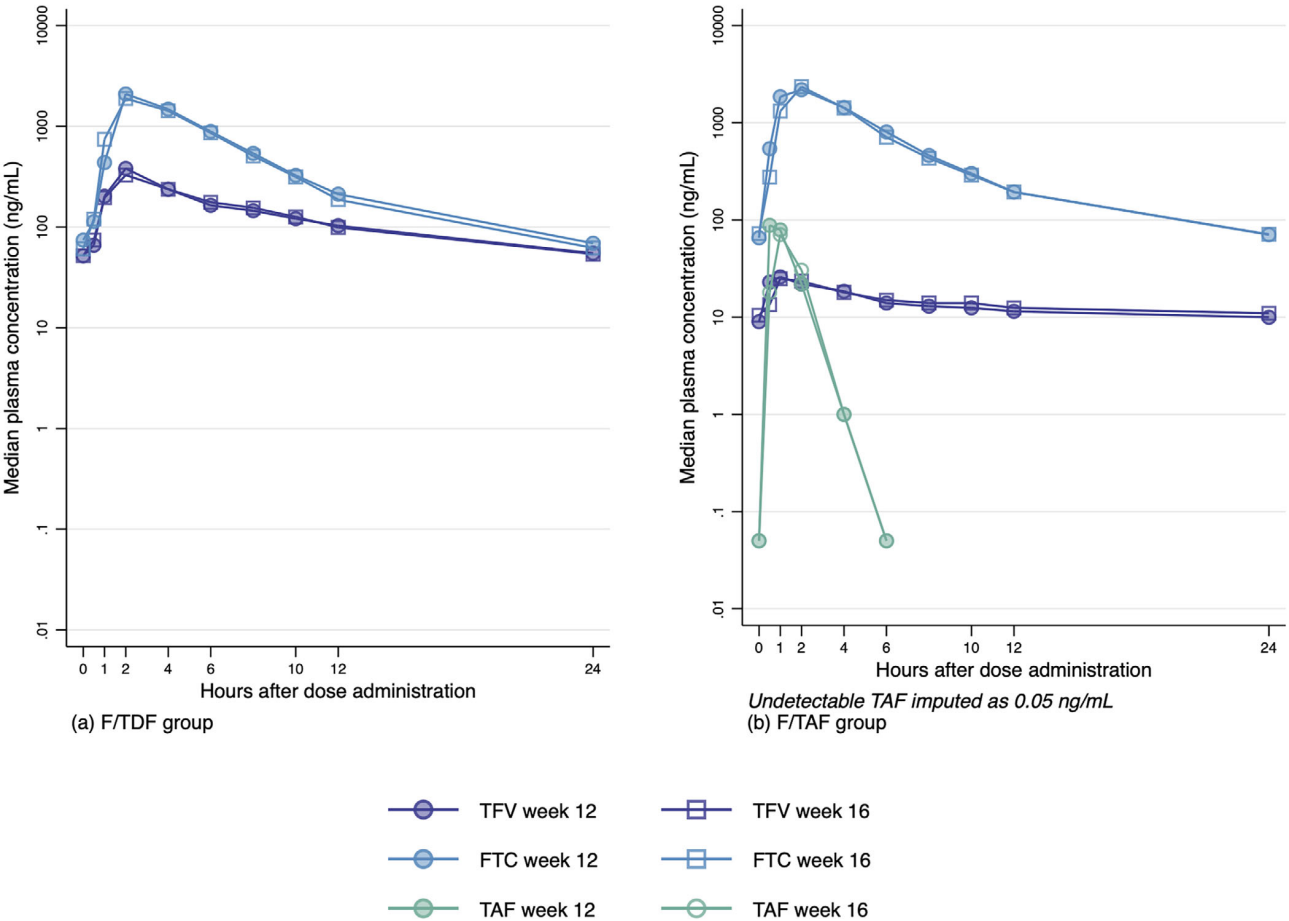
Median concentration times curves of plasma TFV, FTC and TAF (for F/TAF group) at week 12 (with MHT) and week 16 (without MHT): (a) F/TDF group and (b) F/TAF group. Abbreviations: FTC, emtricitabine; MHT, masculinizing hormone therapy; TAF, tenofovir alafenamide; TFV, tenofovir.

The GMRs for AUC_0−24_ and C_max_ at weeks 12 and 16 (reference) for plasma TFV were as follows: F/TDF group, 1.03 (0.96−1.11, *p* = 0.55) and 1.17 (1.03−1.32, *p* = 0.08); and F/TAF group, 1.03 (0.94−1.14, *p* = 0.63) and 1.10 (0.84−1.43, *p* = 0.64), respectively. The GMRs for AUC_0−24_ and C_max_ for plasma FTC were as follows: F/TDF group, 1.05 (1.00−1.09, *p* = 0.12) and 1.09 (1.00−1.18, *p* = 0.17); and F/TAF group, 1.02 (0.99−1.05, *p* = 0.48) and 0.97 (0.85−1.12, *p* = 0.80), respectively. The GMRs for AUC_0−24_ and C_max_ for plasma TAF were 0.99 (0.80−1.21, *p* = 0.93) and 1.00 (0.69−1.44, *p* = 0.99), respectively (Table 2). No significant differences were observed in plasma TAF, TFV and FTC when PrEP was administered with or without MHT. A summary of median plasma concentration‐time profiles of PrEP drug components is presented in Figure 3.

**Table 2:** Geometric mean (%CV) pharmacokinetic parameters for plasma TAF, TFV and FTC

ARV PK parameter	Week 12 (with MHT)	Week 16 (without MHT)	GMR (90% CI)	*p*‐value
**F/TDF group**				
TFV				
AUC_0−24_ (ng*h/ml)	3150.74 (19.18)	3051.46 (28.32)	1.03 (0.96–1.11)	0.55
C_max_ (ng/ml)	430.20 (32.97)	369.03 (39.73)	1.17 (1.03–1.32)	0.08
FTC				
AUC_0−24_ (ng*h/ml)	11,900.06 (14.59)	11,382.04 (17.10)	1.05 (1.00–1.09)	0.12
C_max_ (ng/ml)	2166.22 (16.03)	1993.59 (21.80)	1.09 (1.00–1.18)	0.17
**F/TAF group**				
TAF				
AUC_0−24_ (ng*h/ml)	167.46 (66.84)	169.76 (52.16)	0.99 (0.80–1.21)	0.93
C_max_ (ng/ml)	126.82 (111.11)	127.4 (66.24)	1.00 (0.69–1.44)	0.99
TFV				
AUC_0−24_ (ng*h/ml)	334.85 (44.45)	323.63 (35.35)	1.03 (0.94–1.14)	0.63
C_max_ (ng/ml)	41.25 (75.61)	37.62 (33.72)	1.10 (0.84–1.43)	0.64
FTC				
AUC_0−24_ (ng*h/ml)	13,467.06 (15.42)	13,253.39 (13.17)	1.02 (0.99–1.05)	0.48
C_max_ (ng/ml)	2644.14 (31.27)	2717.64 (26.57)	0.97 (0.85–1.12)	0.80

Abbreviations: %CV, percentage coefficient of variation; ARV, antiretroviral; AUC_0–24_, area under curve from time 0 to 24 hours; CI, confidence interval; C_max_, maximum concentration; FTC, emtricitabine; GMR, geometric mean ratio; MHT, masculinizing hormone therapy; TAF, tenofovir alafenamide; TFV, tenofovir; PK, pharmacokinetic.

### 3.3 Urine TFV and FTC

The GMRs for urine TFV and FTC at week 12 and week 16 were 1.13 (1.00−1.27, *p* = 0.15) and 1.07 (0.97−1.20, *p* = 0.35) for the F/TDF group; and 0.95 (0.83−1.10, *p* = 0.65) and 0.98 (0.86−1.12, *p* = 0.83) for the F/TAF group, respectively. No statistically significant differences were found in urine TFV and FTC concentrations with and without MHT in both groups.

### 3.4 PBMC TFV‐DP and FTC‐TP

The PBMC C_24_ GMRs at week 12 and week 16 for TFV‐DP and FTC‐TP were as follows: F/TDF group, 0.84 (0.57−1.25, *p* = 0.56) and 0.99 (0.81−1.21, *p* = 0.96); F/TAF group, 1.07 (0.67−1.73, *p* = 0.84) and 1.07 (0.84−1.38, *p* = 0.71), respectively. No statistically significant differences were observed in the PK parameters of TFV‐DP and FTC‐TP concentrations in PBMC between the 2 weeks in both groups (Table 3). All participants from both groups achieved TFV‐DP concentrations above the 90% HIV prevention efficacy threshold (EC_90_) of >16 fmol/10^6^ PBMCs at both weeks [19].

**Table 3:** Geometric mean (%CV) pharmacokinetic parameters for PBMC TFV‐DP and FTC‐TP

ARV PK parameter (fmol/million cells)	Week 12 (with MHT)	Week 16 (without MHT)	GMR (90% CI)	*p*‐value
**F/TDF group**				
TFV‐DP				
C_2_	83.00 (79.43)	84.34 (46.96)	0.98 (0.70–1.38)	0.95
C_24_	75.67 (46.61)	86.89 (93.84)	0.84 (0.57–1.25)	0.56
FTC‐TP				
C_2_	4209.88 (61.34)	3282.62 (50.70)	1.17 (0.86–1.60)	0.48
C_24_	3585.09 (87.94)	3307.47 (68.03)	0.99 (0.81–1.21)	0.96
**F/TAF group**				
TFV‐DP				
C_2_	598.85 (99.24)	488.95 (82.14)	1.22 (0.97–1.55)	0.23
C_24_	474.16 (126.88)	440.87 (109.79)	1.07 (0.67–1.73)	0.84
FTC‐TP				
C_2_	5206.26 (93.26)	4731.18 (70.15)	1.10 (0.86–1.41)	0.60
C_24_	3569.09 (63.86)	3335.37 (78.91)	1.07 (0.84–1.38)	0.71

Abbreviations: %CV, percentage coefficient of variation; ARV, antiretroviral; C_2_, concentration at 2 hours; C_24_, concentration at 24 hours; CI, confidence interval; FTC‐TP, emtricitabine‐triphosphate; GMR, geometric mean ratio; MHT, masculinizing hormone therapy; TFV‐DP, tenofovir‐diphosphate; PK, pharmacokinetic.

### 3.5 Rectal and cervical tissue TFV‐DP and FTC‐TP

Among the 20 participants (10 from each group) who underwent rectal and cervical tissue biopsies, no statistically significant differences were observed in TFV‐DP and FTC‐TP concentrations in rectal tissue between week 12 and week 16 in both groups (Table 4). All participants had quantifiable TFV‐DP concentrations from rectal tissues. However, there were statistically significant differences in TDF‐DP and FTC‐TP concentrations in cervical tissue between week 12 and week 16 in the F/TAF group. TFV‐DP increased from 12.9 [6.78–14.56] fmol/mg at week 12 to 20.63 [7.47–53.43] fmol/mg at week 16 (*p* = 0.04), and FTC‐TP increased from 67.05 [27.24–77.24] fmol/mg to 120.43 [65.98–245.76] fmol/mg (*p* = 0.02). All but one participant (from the F/TDF group) had quantifiable TFV‐DP concentrations from cervical tissues. No significant differences in both active metabolites in cervical tissue for the F/TDF group were observed.

**Table 4:** Median (IQR) pharmacokinetic parameters for cervical and rectal tissue TFV‐DP and FTC‐TP (*N* = 20)

ARV PK parameter (fmol/mg)	Week 12 (with MHT)	Week 16 (without MHT)	*p*‐value
**F/TDF group (*N* = 10)**			
*Cervical tissue*			
TFV‐DP	8.70 (3.96–12.38)	5.86 (2.75–9.10)	0.51
FTC‐TP	30.25 (5.10–143.04)	34.02 (28.96–153.75)	0.28
*Rectal tissue*			
TFV‐DP	211.74 (82.36–517.72)	61 (33.11–388.71)	0.65
FTC‐TP	8.33 (4.94–11.88)	5.23 (4.39–7.16)	0.11
**F/TAF group (*N* = 10)**			
*Cervical tissue*			
TFV‐DP	12.90 (6.78–14.56)	20.63 (7.47–53.43)	0.04
FTC‐TP	67.05 (27.24–77.24)	120.43 (65.98–245.76)	0.02
*Rectal tissue*			
TFV‐DP	53.42 (30.30–185.37)	65.31 (32.80–92.30)	0.51
FTC‐TP	7.60 (6.40–14.68)	7.70 (5.60–11.09)	0.51

Abbreviations: ARV, antiretroviral; FTC‐TP, emtricitabine‐triphosphate; IQR, interquartile range; MHT, masculinizing hormone therapy; PK, pharmacokinetic; TFV‐DP, tenofovir‐diphosphate.

### 3.6 Comparisons between F/TDF and F/TAF groups

Plasma TFV AUC_0−24_ and C_max_ in the F/TDF group were significantly higher than the F/TAF group by 9–10 fold, while PBMC TFV‐DP concentrations were significantly lower by 80–86% (Table 5). Plasma FTC PK parameters in the F/TDF group were slightly but significantly lower by 12–27%. Median cervical TFV‐DP concentrations in the F/TDF group were significantly lower than F/TAF in week 16 (5.86 [2.75−9.10] vs. 20.63 [7.47−53.43] fmol/mg, *p* = 0.01). There were no significant differences in rectal tissue TFV‐DP and FTC‐TP concentrations between the two groups.

**Table 5:** Comparisons of geometric mean (%CV) pharmacokinetic parameters for PrEP drug components from plasma, PBMC, and cervical and rectal tissues

	F/TDF group (*N* = 19)	F/TAF group (*N* = 20)	GMR (95% CI)	*p*‐value
**Week 12 (with MHT)**				
Plasma TFV				
AUC_0−24_ (ng*h/ml)	3150.74 (19.18)	334.85 (44.45)	9.41 (7.61–11.63)	<0.001
C_max_ (ng/ml)	430.20 (32.97)	41.25 (75.61)	10.43 (7.43–14.64)	<0.001
Plasma FTC				
AUC_0−24_ (ng*h/ml)	11,900.06 (14.59)	13,467.06 (15.42)	0.88 (0.80–0.97)	0.01
C_max_ (ng/ml)	2166.22 (16.03)	2644.14 (31.27)	0.82 (0.70–0.96)	0.01
PBMC TFV (fmol/10^6^ cells)				
C_2_	83.00 (79.43)	598.85 (99.24)	0.14 (0.08–0.23)	<0.001
C_24_	75.67 (46.61)	474.16 (126.88)	0.16 (0.10–0.26)	<0.001
PBMC FTC‐TP (fmol/10^6^ cells)				
C_2_	4209.88 (61.34)	5206.26 (93.26)	0.81 (0.52–1.27)	0.34
C_24_	3585.09 (87.94)	3569.09 (63.86)	0.92 (0.65–1.31)	0.63
Cervical tissue (fmol/mg)				
TFV‐DP	8.70 (3.96–12.38)	12.90 (6.78–14.56)		0.26
FTC‐TP	30.25 (5.10–143.04)	67.05 (27.24–77.24)		0.65
Rectal tissue (fmol/mg)				
TFV‐DP	211.74 (82.36–517.72)	53.42 (30.30–185.37)		0.07
FTC‐TP	8.33 (4.94–11.88)	7.60 (6.40–14.68)		0.88
**Week 16 (without MHT)**				
Plasma TFV				
AUC_0−24_ (ng*h/ml)	3051.46 (28.32)	323.63 (35.35)	9.42 (7.70–11.54)	<0.001
C_max_ (ng/ml)	369.03 (39.73)	37.62 (33.72)	9.81 (7.78–12.37)	<0.001
Plasma FTC				
AUC_0−24_ (ng*h/ml)	11,382.04 (17.10)	13,253.39 (13.17)	0.86 (0.78–0.95)	0.003
C_max_ (ng/ml)	1993.59 (21.80)	2717.64 (26.57)	0.73 (0.63–0.86)	<0.001
PBMC TFV (fmol/fmol/10^6^ cells)				
C_2_	84.34 (46.96)	488.95 (82.14)	0.17 (0.12–0.26)	<0.001
C_24_	86.89 (93.84)	440.87 (109.79)	0.20 (0.11–0.35)	<0.001
PBMC FTC‐TP (fmol/fmol/10^6^ cells)				
C_2_	3282.62 (50.70)	4731.18 (70.15)	0.76 (0.48–1.19)	0.22
C_24_	3307.47 (68.03)	3335.37 (78.91)	0.99 (0.65–1.52)	0.97
Cervical tissue (fmol/mg)				
TFV‐DP	5.86 (2.75–9.10)	20.63 (7.47–53.43)		0.01
FTC‐TP	34.02 (28.96–153.75)	120.43 (65.98–245.76)		0.13
Rectal tissue (fmol/mg)				
TFV‐DP	61 (33.11–388.71)	65.31 (32.80–92.30)		0.45
FTC‐TP	5.23 (4.39–7.16)	7.70 (5.60–11.09)		0.20

*Note*: Data on cervical and rectal tissue PK parameters are presented as median (IQR).

Abbreviations: %CV, percentage coefficient of variation; ARV, antiretroviral; AUC_0–24_, area under curve from time 0 to 24 hours; C_2_, concentration at 2 hours; C_24_, concentration at 24 hours; CI, confidence interval; C_max_, maximum concentration; FTC‐TP, emtricitabine‐triphosphate; GMR, geometric mean ratio; MHT, masculinizing hormone therapy; PK, pharmacokinetic; TFV‐DP, tenofovir‐diphosphate.

### 3.7 Safety

There was a significant decrease in the median CrCl from baseline to week 6 in both groups (F/TDF: 124.9 [105.1–138.5] to 99.3 [91.1–114.8], *p* = 0.001; and F/TAF: 123.7 [102.0–145.8] to 97.6 [85.9–118.4], *p* < 0.001). The CrCl remained stable from week 6 until the end of the study. There were no significant changes in ALT levels throughout the study period. No participants acquired HIV.

## 4 DISCUSSION

Our PK study assessed the potential impact of daily oral F/TDF‐based PrEP and F/TAF‐based PrEP on MHT, and vice versa, among Thai transgender men. No significant changes were observed in the PK of plasma total testosterone when MHT was administered with or without either F/TDF or F/TAF PrEP. Similarly, there were no significant changes in the PK of plasma TFV, FTC and TAF (for the F/TAF group); urine TFV and FTC; and PBMC and rectal tissue TFV‐DP and FTC‐TP when PrEP was administered with or without MHT. Notably, both TFV‐DP and FTC‐TP concentrations in cervical tissue were significantly lower when F/TAF‐based PrEP was administered with MHT. Concerns regarding potential DDIs between GAHT and PrEP have been raised as key barriers to the uptake and adherence of PrEP among transgender persons [3]. However, given the distinct metabolic pathways of F/TDF and F/TAF components and MHT, clinically significant DDIs are not anticipated.

Previous studies, all conducted in the United States, explored the potential DDIs from F/TDF‐based PrEP towards MHT [10, 12, 13]. Among these, one study was a PK study comparing baseline and after 2–3 weeks of daily F/TDF, showing not significant changes in total testosterone PK parameters [13]. Our study aligns with these findings, as no significant differences in total testosterone PK levels were observed with or without the concomitant use of daily oral PrEP. This further supports that F/TDF does not significantly affect MHT. Additionally, our study provides new evidence showing that similar results occur with F/TAF.

Two previous studies investigated the potential impact of MHT on F/TDF‐based PrEP. In one study, a comparison of TDF‐DP concentrations in DBS from transgender men who used (*n* = 39) and did not use MHT (*n* = 10) after 12 weeks of taking F/TDF showed no significant differences [12]. Another study, also using DBS, assessed TFV‐DP and FTC‐TP concentrations in 24 transgender men after 4 weeks of F/TDF use, showing comparable TFV‐DP concentrations with transgender women but lower concentrations compared to cisgender women (mean difference −23% [95% CI: −36% to −7%], *p* = 0.007). However, all transgender men were projected to achieve TFV‐DP concentrations associated with high protection against HIV acquisition [10]. We found no significant differences in PK parameters of both F/TDF and F/TAF in multiple anatomical compartments (i.e. plasma, urine, PBMC and rectal tissue) when PrEP was administered with or without MHT. Importantly, PBMC TFV‐DP concentrations exceeded the EC_90_ of >40 fmol/10^6^ PBMCs at both visits, complementing previous studies from other anatomical compartments.

CD4‐bearing cells in cervical tissues play a key role in HIV transmission during vaginal intercourse. The female genital tract has lower drug concentrations than rectal tissue following F/TDF, raising concerns for HIV prevention. While TAF achieves higher intracellular TFV‐DP levels in PBMCs than TDF, this advantage is inconsistent in vaginal and cervical tissues, with significantly lower or undetectable TFV‐DP levels compared to TDF [17, 20, 21]. Consequently, F/TAF‐based regimens are not currently recommended for persons assigned female at birth, including transgender men. Our study found that while TFV‐DP concentrations in cervical tissues remained similar with F/TDF‐based PrEP regardless of MHT use, they were significantly lower in those taking F/TAF‐based PrEP with MHT compared to those without MHT. Further studies are needed to explore the underlying mechanism behind this reduction when F/TAF was used, establish optimal cervical tissue target levels for HIV prevention, and assess levels in vaginal tissue and fluid among transgender men.

Certain limitations need to be considered in our study. First, we did not employ a randomized crossover design, which could introduce a potential period effect. However, the study drugs’ half‐lives and the intervals between intensive PK sampling ensured that steady‐state conditions are achieved for each drug of interest, and that elimination of the drug dosed in the prior period was completed. Issues related to PrEP adherence, as well as concurrent use of other MHT, are possible. Nevertheless, we provided our best effort to ensure protocol adherence by directly observing PrEP administration and confirmed the use of other concurrent medications at every study visit. The use of a specific MHT regimen in this study may limit the generalizability of our findings to those using different MHT regimens. Another limitation is that participants in this study had cervical tissue with recent exposure to MHT, which may differ from the cervical tissue of transgender men who have been using testosterone for a longer period. Lastly, the nature of an intensive PK study design meant that all participants enrolled in our study had highly homogenous characteristics. Therefore, we cannot extrapolate conclusions regarding the presence of any DDIs between MHT and oral PrEP to transgender men with different ethnicities, races, ages or BMI from those of our study participants; although this was not the case for F/TDF among transgender women [6, 7, 8].

## 5 CONCLUSIONS

Plasma total testosterone concentration remained unchanged in transgender males on MHT in the presence of F/TDF‐ or F/TAF‐based PrEP. Plasma and urine PK parameters, as well as intracellular TFV‐DP and FTC‐TP concentrations in PBMC and rectal tissue, were comparable when F/TDF‐ or F/TAF‐based PrEP was administered with or without MHT. TFV‐DP and FTC‐TP concentrations trended significantly lower in cervical tissue when F/TAF‐based PrEP was administered with MHT. The findings suggest that oral PrEP should not affect the effectiveness of MHT and that F/TDF‐based PrEP should be effective when taken with MHT. However, further research is needed to assess the effectiveness of TAF‐based PrEP in transgender men.

## COMPETING INTERESTS

All authors declare no competing interests related to this work.

## AUTHORS’ CONTRIBUTIONS

AH drafted the manuscript and study protocol. AH and NP initiated the study concept and led the study. NT and SN coordinated the study operations. AH, SK and RJ contributed to the study design. RJ and NP facilitated the grant coordination for the study. AH and SK conducted all statistical analyses. RT, YT, TRC and PLA performed the laboratory testing. NT, PH, JB and NT oversaw the participants. All authors critically reviewed and approved the final draft of the manuscript.

## FUNDING

This study was supported by an Investigator Sponsored Research (ISR) grant through Gilead Sciences (IN‐US‐412‐5796).

## Supporting information




**Supplementary File**. Details of specimen collection and processing procedure

## Data Availability

The data that support the findings of this study are available from the corresponding author upon reasonable request.

## References

[1] Huang YA , Radix A , Zhu W , Kimball AA , Olansky EJ , Hoover KW . HIV testing and preexposure prophylaxis prescriptions among U.S. commercially insured transgender men and women, 2014 to 2021. Ann Intern Med. 2024;177(1):12–7. 38109739 10.7326/M23-2073

[2] Sevelius JM , Keatley J , Calma N , Arnold E . ‘I am not a man’: trans‐specific barriers and facilitators to PrEP acceptability among transgender women. Glob Public Health. 2016;11(7–8):1060–75. 26963756 10.1080/17441692.2016.1154085 PMC10204128

[3] Braun HM , Candelario J , Hanlon CL , Segura ER , Clark JL , Currier JS , et al. Transgender women living with HIV frequently take antiretroviral therapy and/or feminizing hormone therapy differently than prescribed due to drug−drug interaction concerns. LGBT Health. 2017;4(5):371–5. 28876170 10.1089/lgbt.2017.0057 PMC5661861

[4] Coleman E , Bockting W , Botzer M , Cohen‐Kettenis P , DeCuypere G , Feldman J , et al. Standards of care (SOC) for the health of transsexual, transgender, and gender nonconforming people. Georgia: World Professional Association Transgender Health; 2011.

[5] Radix A , Sevelius J , Deutsch MB . Transgender women, hormonal therapy and HIV treatment: a comprehensive review of the literature and recommendations for best practices. J Int AIDS Soc. 2016;19(3Suppl 2):20810. 27431475 10.7448/IAS.19.3.20810 PMC4949308

[6] Hiransuthikul A , Janamnuaysook R , Himmad K , Kerr SJ , Thammajaruk N , Pankam T , et al. Drug−drug interactions between feminizing hormone therapy and pre‐exposure prophylaxis among transgender women: the iFACT study. J Int AIDS Soc. 2019;22(7):e25338. 31298497 10.1002/jia2.25338 PMC6625338

[7] Cottrell ML , Prince HMA , Schauer AP , Sykes C , Maffuid K , Poliseno A , et al. Decreased tenofovir diphosphate concentrations in a transgender female cohort: implications for human immunodeficiency virus preexposure prophylaxis. Clin Infect Dis. 2019;69(12):2201–4. 30963179 10.1093/cid/ciz290 PMC7188232

[8] Shieh E , Marzinke MA , Fuchs EJ , Hamlin A , Bakshi R , Aung W , et al. Transgender women on oral HIV pre‐exposure prophylaxis have significantly lower tenofovir and emtricitabine concentrations when also taking oestrogen when compared to cisgender men. J Int AIDS Soc. 2019;22(11):e25405. 31692269 10.1002/jia2.25405 PMC6832671

[9] Cirrincione LR , Podany AT , Havens JP , Bares SH , Dyavar SR , Gwon Y , et al. Plasma and intracellular pharmacokinetics of tenofovir disoproxil fumarate and emtricitabine in transgender women receiving feminizing hormone therapy. J Antimicrob Chemother. 2020;75(5):1242–9. 32065631 10.1093/jac/dkaa016 PMC7177476

[10] Grant RM , Pellegrini M , Defechereux PA , Anderson PL , Yu M , Glidden DV , et al. Sex hormone therapy and tenofovir diphosphate concentration in dried blood spots: primary results of the interactions between antiretrovirals and transgender hormones study. Clin Infect Dis. 2021;73(7):e2117–e23. 32766890 10.1093/cid/ciaa1160 PMC8492111

[11] Cespedes MS , Das M , Yager J , Prins M , Krznaric I , de Jong J , et al. Gender affirming hormones do not affect the exposure and efficacy of F/TDF or F/TAF for HIV preexposure prophylaxis: a subgroup analysis from the DISCOVER Trial. Transgender Health. 2024;9(1):46–52. 38312459 10.1089/trgh.2022.0048 PMC10835152

[12] Blumenthal J , Goyal R , Burke L , Dubé M , Hoenigl M , Moore DJ , et al. The bi‐directional effects of hormone therapy and PrEP in transgender individuals (Abstract #84). Conference on Retroviruses and Opportunistic Infections (CROI). 2022.

[13] Yager J , Brooks KM , Brothers J , Mulligan K , Landovitz RJ , Reirden D , et al. Gender‐affirming hormone pharmacokinetics among adolescent and young adult transgender persons receiving daily emtricitabine/tenofovir disoproxil fumarate. AIDS Res Hum Retroviruses. 2022;38(12):939–43. 35815468 10.1089/aid.2022.0044 PMC9910105

[14] Cattani VB , Jalil EM , Eksterman L , Torres T , Wagner Cardoso S , Castro CRV , et al. Estradiol and spironolactone plasma pharmacokinetics among Brazilian transgender women using HIV pre‐exposure prophylaxis: analysis of potential interactions. Clin Pharmacokinet. 2023;62(7):1031–41. 37261664 10.1007/s40262-023-01248-0 PMC10338392

[15] Podany AT , Bares SH , Havens J , Dyavar SR , O'Neill J , Lee S , et al. Plasma and intracellular pharmacokinetics of tenofovir in patients switched from tenofovir disoproxil fumarate to tenofovir alafenamide. AIDS. 2018;32(6):761–5. 29334548 10.1097/QAD.0000000000001744 PMC5854526

[16] Mayer KH , Molina JM , Thompson MA , Anderson PL , Mounzer KC , De Wet JJ , et al. Emtricitabine and tenofovir alafenamide vs emtricitabine and tenofovir disoproxil fumarate for HIV pre‐exposure prophylaxis (DISCOVER): primary results from a randomised, double‐blind, multicentre, active‐controlled, phase 3, non‐inferiority trial. Lancet. 2020;396(10246):239–54. 32711800 10.1016/S0140-6736(20)31065-5 PMC9665936

[17] Thurman AR , Schwartz JL , Cottrell ML , Brache V , Chen BA , Cochón L , et al. Safety and pharmacokinetics of a tenofovir alafenamide fumarate‐emtricitabine based oral antiretroviral regimen for prevention of HIV acquisition in women: a randomized controlled trial. EClinicalMedicine. 2021;36:100893. 34041459 10.1016/j.eclinm.2021.100893 PMC8144741

[18] Hembree WC , Cohen‐Kettenis PT , Gooren L , Hannema SE , Meyer WJ , Murad MH , et al. Endocrine treatment of gender‐dysphoric/gender‐incongruent persons: an Endocrine Society* Clinical Practice Guideline. J Clin Endocrinol Metab. 2017;102(11):3869–903. 28945902 10.1210/jc.2017-01658

[19] Anderson PL , Glidden DV , Liu A , Buchbinder S , Lama JR , Guanira JV , et al. Emtricitabine‐tenofovir concentrations and pre‐exposure prophylaxis efficacy in men who have sex with men. Sci Transl Med. 2012;4(151):151ra25. 10.1126/scitranslmed.3004006 PMC3721979 22972843

[20] Cottrell ML , Garrett KL , Prince HMA , Sykes C , Schauer A , Emerson CW , et al. Single‐dose pharmacokinetics of tenofovir alafenamide and its active metabolite in the mucosal tissues. J Antimicrob Chemother. 2017;72(6):1731–40. 28369415 10.1093/jac/dkx064 PMC5536328

[21] Cottrell ML , Yang KH , Prince HM , Sykes C , White N , Malone S , et al. A translational pharmacology approach to predicting outcomes of preexposure prophylaxis against HIV in men and women using tenofovir disoproxil fumarate with or without emtricitabine. J Infect Dis. 2016;214(1):55–64. 26917574 10.1093/infdis/jiw077 PMC4907409

